# Supplementary material for: Chronos: a cell population dynamics model of CRISPR experiments that improves inference of gene fitness effects
Source: Genome Biol. 2021 Dec 20;22:343. doi: 10.1186/s13059-021-02540-7 (PMC8686573; doi:10.1186/s13059-021-02540-7)
Supplement: Supplementary file 1 — Additional file 1: Supplementary figures. [file 13059_2021_2540_MOESM1_ESM.pdf]

Chronos: a cell population dynamics model of CRISPR experiments that improves inference of gene fitness effects

Joshua M. Dempster[1], Isabella Boyle[1], Francisca Vazquez[1], David E. Root[1], Jesse S. Boehm[1], William C. Hahn[1,2], Aviad Tsherniak[1], James M. McFarland[1*]

[1] Broad Institute of MIT and Harvard, 415 Main Street, Cambridge, MA 02142, USA
[2] Dana-Farber Cancer Institute, 450 Brookline Ave, Boston, MA 02215, USA
[*] Corresponding author

ADDITIONAL FILE 1: SUPPLEMENTARY FIGURES

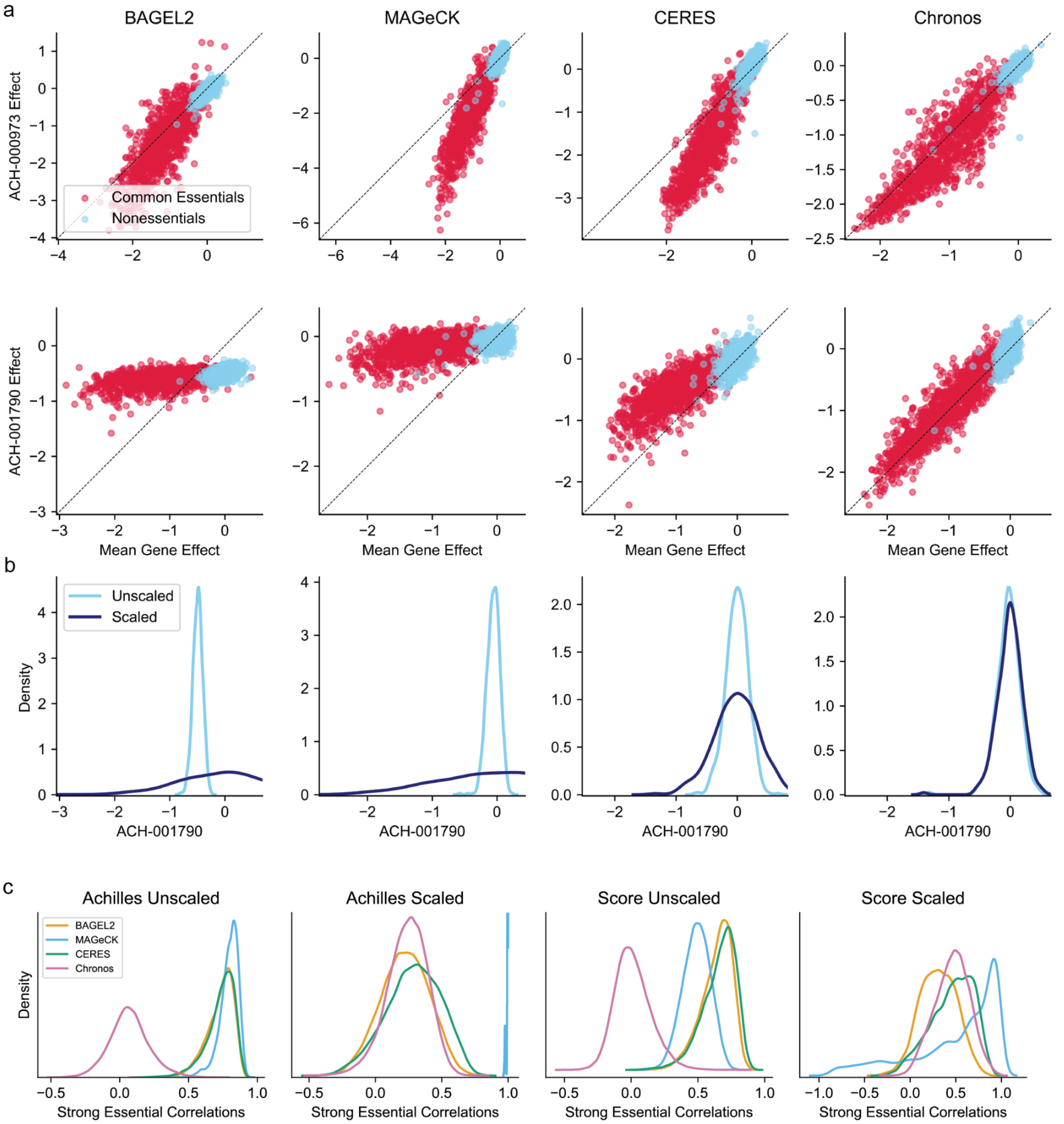

Figure S1: Screen quality bias. a. Unscaled gene fitness effects for common essential and nonessential genes in an Achilles screen with good quality (top) and poor quality (bottom), plotted against the average gene fitness effects for those genes across cell lines. Each point is a gene. **b.** A simple strategy to reduce screen quality bias is to shift and scale each screen such that the median of common essentials is the same value (-1 here). However, this can dramatically expand the noise in low quality screens. The two distributions show the width of the nonessential gene fitness effects in ACH-001790 (RH18-DM) before and after scaling by common essentials. **c.** Mutual correlations between gene effects for all possible pairings of the 300 strongest common essentials (most negative mean gene effect) for each version of the data, before and after scaling. The near-unity correlations for scaled MAGeCK strong essentials in Achilles data is driven by the extreme inflation of a few cell lines with almost no signal under MAGeCK processing.

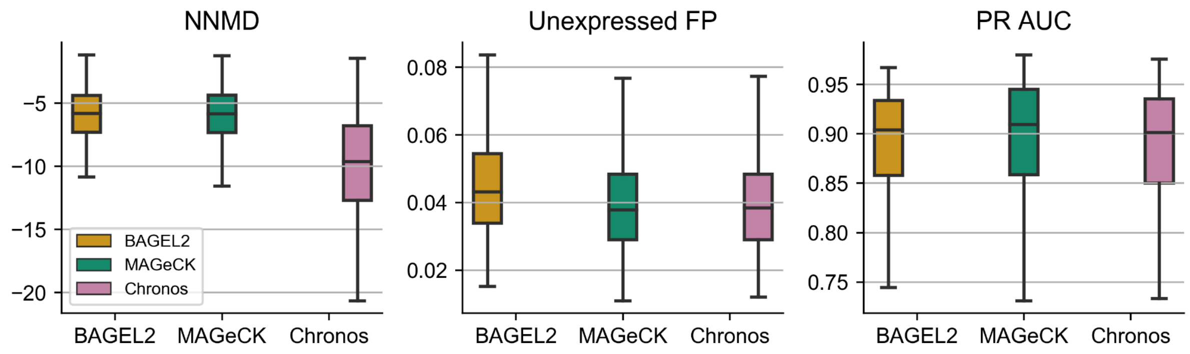

Figure S2: Control separation with single-line runs. a. NNMD separation of common essential and unexpressed genes in Achilles screens run individually in each algorithm. **b.** Similar for the number of unexpressed false positives. **c**. Similar for the area under the precision-recall curve.

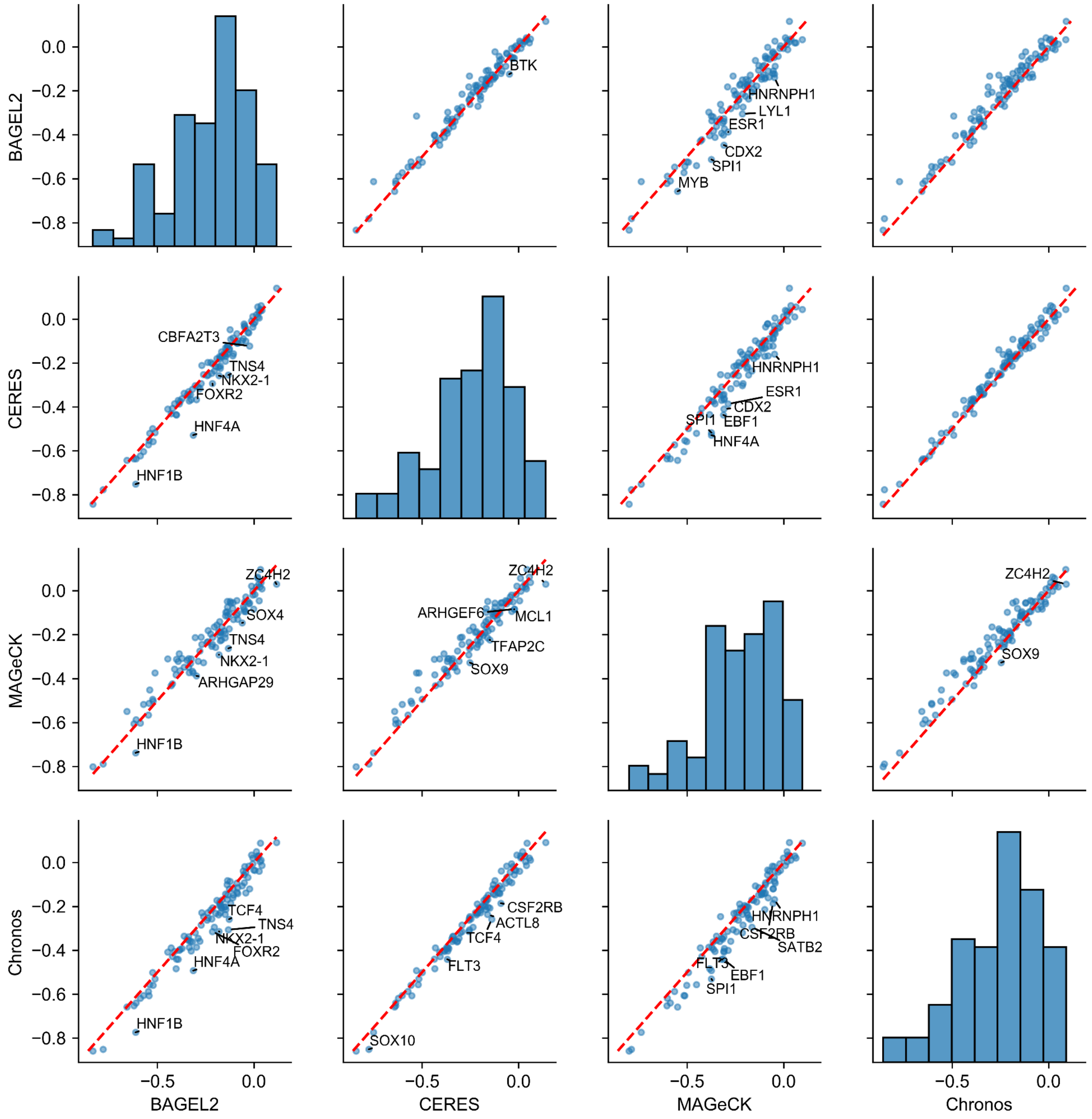

Figure S3: Comparison of Expression Addictions in Achilles. For RNAi-discovered expression addictions, the correlation of the gene's gene effect with its own expression as estimated by the different algorithms. Genes are labeled if their correlation in the x-axis algorithm is at least 0.06 more negative than in the y-axis algorithm, up to the first six such genes.

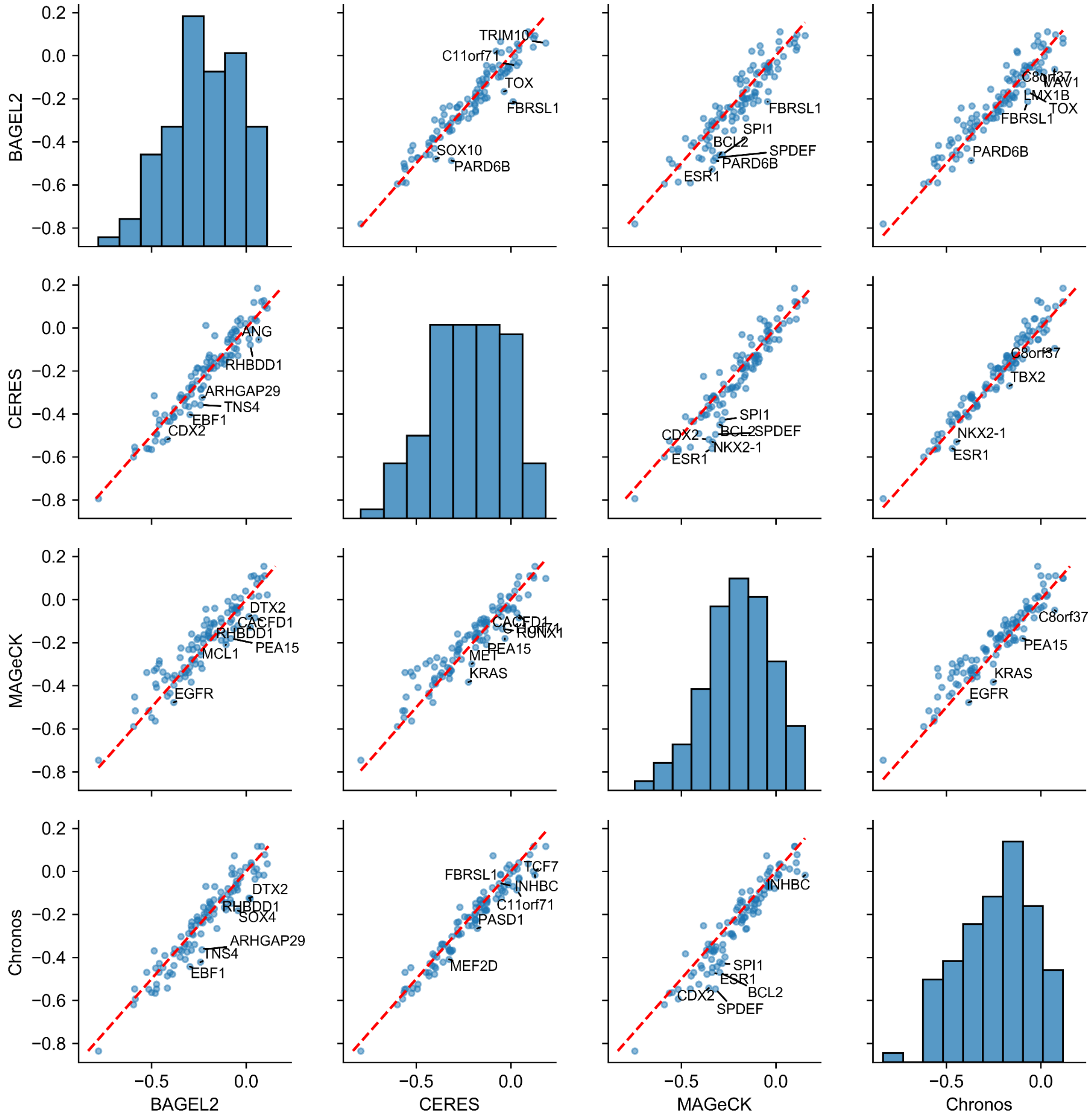

Figure S4: Comparison of Expression Addictions in Score. As in Supplementary Fig. 3, using Project Score data.

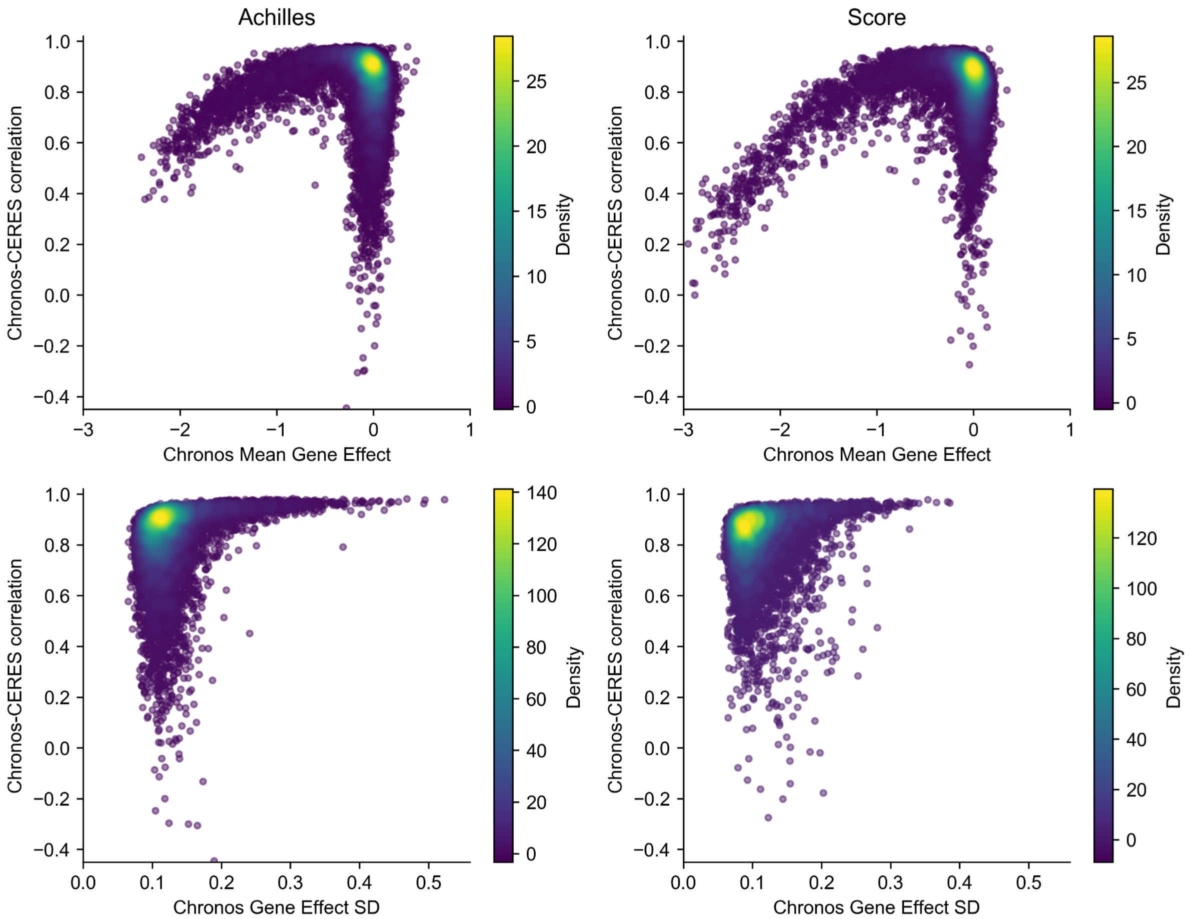

Fig. S5: Influence of gene effect mean and standard deviation on Chronos-CERES correlation. Correlation of gene effect profiles for each shared gene between Chronos and CERES estimates vs Chronos's mean effect estimate for that gene (top) and standard deviation (bottom). Agreement between the two methods is lowest for the genes with strongest average gene effect, and is highly variable for genes with low standard deviation..
